# Lutein and Zeaxanthin Enhance, Whereas Oxidation, Fructosylation, and Low pH Damage High-Density Lipoprotein Biological Functionality

**DOI:** 10.3390/antiox13050616

**Published:** 2024-05-18

**Authors:** Jingyuan Zheng, Brian V. Hong, Joanne K. Agus, Xinyu Tang, Nola R. Klebaner, Siyu Chen, Fei Guo, Danielle J. Harvey, Carlito B. Lebrilla, Angela M. Zivkovic

**Affiliations:** 1 Department of Nutrition, University of California Davis, Davis, CA 95616, USA; jaczheng@ucdavis.edu (J.Z.); bvhong@ucdavis.edu (B.V.H.); jkagus@ucdavis.edu (J.K.A.); xctang@ucdavis.edu (X.T.); nrklebaner@ucdavis.edu (N.R.K.); 2 Department of Chemistry, University of California Davis, Davis, CA 95616, USA; siych@ucdavis.edu (S.C.); cblebrilla@ucdavis.edu (C.B.L.); 3 Department of Molecular and Cellular Biology, University of California Davis, Davis, CA 95616, USA; feiguo@ucdavis.edu; 4 Department of Public Health Sciences, University of California Davis, Davis, CA 95616, USA; djharvey@ucdavis.edu

**Keywords:** HDL, carotenoids, chronic inflammation, oxidative stress

## Abstract

High-density lipoproteins (HDLs) are key regulators of cellular cholesterol homeostasis but are functionally altered in many chronic diseases. The factors that cause HDL functional loss in chronic disease are not fully understood. It is also unknown what roles antioxidant carotenoids play in protecting HDL against functional loss. The aim of this study was to measure how various disease-associated chemical factors including exposure to (1) Cu^2+^ ions, (2) hypochlorous acid (HOCL), (3) hydrogen peroxide (H_2_O_2_), (4) sialidase, (5) glycosidase, (6) high glucose, (7) high fructose, and (8) acidic pH, and the carotenoid antioxidants (9) lutein and (10) zeaxanthin affect HDL functionality. We hypothesized that some of the modifications would have stronger impacts on HDL particle structure and function than others and that lutein and zeaxanthin would improve HDL function. HDL samples were isolated from generally healthy human plasma and incubated with the corresponding treatments listed above. Cholesterol efflux capacity (CEC), lecithin–cholesterol acyl transferase (LCAT) activity, and paraoxonase-1 (PON1) activity were measured in order to determine changes in HDL functionality. Median HDL particle diameter was increased by acidic pH treatment and reduced by HOCl, high glucose, high fructose, *N*-glycosidase, and lutein treatments. Acidic pH, oxidation, and fructosylation all reduced HDL CEC, whereas lutein, zeaxanthin, and sialidase treatment improved HDL CEC. LCAT activity was reduced by acidic pH, oxidation, high fructose treatments, and lutein. PON1 activity was reduced by sialidase, glycosidase, H_2_O_2_, and fructose and improved by zeaxanthin and lutein treatment. These results show that exposure to oxidizing agents, high fructose, and low pH directly impairs HDL functionality related to cholesterol efflux and particle maturation, whereas deglycosylation impairs HDL antioxidant capacity. On the other hand, the antioxidants lutein and zeaxanthin improve or preserve both HDL cholesterol efflux and antioxidant activity but have no effect on particle maturation.

## 1. Introduction

Although high-density lipoprotein (HDL) cholesterol (HDL-C) concentration has been found to be associated with protection from several chronic diseases such as cardiovascular disease (CVD) [1,2,3,4] and Alzheimer’s disease (AD) [5,6], HDL-C itself may not be the correct target for improving risk. Some genetic variations that lead to low circulating HDL-C are not associated with higher CVD risk [7,8], and on the other hand, pharmaceutical interventions that raise HDL-C failed to prevent CVD progression [9]. Thus, the focus has turned to understanding and measuring other aspects of HDL, in particular HDL particle size distribution and functionality.

The beneficial properties of HDL particles, including cholesterol efflux capacity (CEC) [10,11], anti-inflammatory [12], and antioxidant functions [12,13], participate in several important biological processes that have been linked with disease development. However, HDL particles can be damaged and modified over time as they circulate before being cleared, especially in pathological states with high levels of oxidative stress, changes in pH, high glucose or fructose concentrations, and other chemical and enzymatic processes observed to occur in chronic disease states.

Oxidation has been found to diminish the antioxidant capacity and CEC of HDL particles, and these dysfunctional, oxidized HDL accumulate in human atheromatous plaque [14,15,16]. Hypochlorous acid released by myeloperoxidase in activated macrophages and the accumulation of metal ions such as Cu^2+^ can oxidize several components in HDL [17]. Glycation of apolipoprotein A-I (apoA-I), the major structural and functional apolipoprotein in HDL, with ribose or glucose resulted in the formation of advanced glycation end products (AGE) and reduction in CEC [18], though this result was not confirmed in another study [19]. HDL particles that are both glycated and oxidized inhibit platelet aggregation and have altered lipid profiles [20]. Alterations in protein glycosylation may also be involved in the loss of function of HDL particles, as demonstrated, for example, by differences in the HDL glycome of patients with vs. without coronary artery disease (CAD) [21] and differences in the immunomodulatory capacity of HDL particles with differential glycoproteomic profiles [22]. Under acidic pH, HDL particles have been found to undergo substantial structural remodeling and functional alterations [23].

Antioxidant molecules such as vitamin E are thought to protect HDL from oxidation. However, vitamin E (α- and γ-tocopherol) was found to actually promote HDL oxidation [24]. Lutein and zeaxanthin are two other antioxidant molecules that are primarily transported by HDL [25,26]. They are the predominant carotenoids of the macular pigment, which protect the retina from light-induced damage and age-related macular degeneration [27]. Several studies showed that lutein/zeaxanthin intake increased HDL-C concentrations in humans and animals [28,29,30]. Yet, their antioxidant impact on HDL functions has not been fully resolved.

A comprehensive, head-to-head comparison of the impact of these different modifications on HDL particle structure and function has not yet been performed. In this study, we used a single pooled HDL sample to directly compare the effects of different chemical modifications on HDL structure and function. We hypothesized that some of the modifications would have stronger impacts on HDL particle structure and function than others and that lutein and zeaxanthin would improve HDL function.

## 2. Materials and Methods

### 2.1. Sample Collection and HDL Isolation

Human fasting plasma was derived from blood samples from twenty men and women aged 18–45 years old at Ragle Human Nutrition Center, University of California, Davis, between April 2019 and December 2019. Donors were generally healthy, did not take any medication, or had any comorbidities that would affect HDL functions. The study was approved by the Institutional Review Board of UC Davis. A detailed description of the participants, exclusion criteria, and study design were described in a previous report [31]. A single plasma pool of 500 μL from each of the 20 participants was generated, and then HDL was isolated using a previously published method [32]. Briefly, 0.5 mL plasma was underlaid beneath 4.2 mL of 1.006 g/mL potassium bromide (KBr) solution (1.095% KBr *w*/*w* in deionized water) in a 4.7 mL OptiSeal Polypropylene Tube (Beckmann-Coulter, Indianapolis, IN, USA). The layered mixture was submitted to ultracentrifugation at 110,000 rpm (657,272× *g*) using a fixed-angle rotor (TLA-110, Beckmann-Coulter, Indianapolis, IN, USA) in a Beckman Optima MAX-TL Ultracentrifuge (Beckmann-Coulter, Indianapolis, IN, USA) for 90 min. The top 4.0 mL solution was removed, and the bottom 0.7 mL solution was mixed with 1.1 mL 1.340 g/mL KBr solution (59.11% KBr *w*/*w* in deionized water) to reach an adjusted density of 1.210 g/mL. The mixture was then carefully underlaid beneath 2.9 mL of 1.210 g/mL KBr solution (9.439% KBr *w*/*w* in deionized water) in a new OptiSeal Polypropylene Tube and submitted to ultracentrifugation at 110,000 rpm (657,272× *g*) using the same rotor and centrifuge for 30 min. A centrifuged solution was retrieved, and the top 2 mL layer containing HDL was obtained, diluted with 2 mL 0.01 M phosphate-buffered saline (1× PBS, Thermo Fisher, Waltham, MA, USA, Cat. No. 10010023), and filtered through a filtering unit (Amicon Ultra-4 50 kDa, MilliporeSigma, Burlington, MA, USA, Cat. No. UFC805024) to remove remaining KBr ions. The filtered solution was resuspended to 0.25 mL and was further separated by particle size using a Superdex 200 Increase 10/300 GL agarose-crosslinked column (GE Healthcare, Chicago, IL, USA) mounted on an HPLC system (1260 Infinity II LC System, Agilent Technology, Santa Clara, CA, USA) with a flowrate of 0.4 mL/min. HDL Fractions were collected at elution volume between 10 mL and 13.6 mL (Agilent 1260 FC-AS, Agilent Technology, Santa Clara, CA, USA). The HDL isolates were pooled together to generate one consistent HDL sample from the plasma pool. Cryoprotectant (2% sucrose) was added to the collected HDL fraction, and samples were stored at −80 °C before use. Isolated HDL samples were quantified for total protein concentration using a commercially available microBCA protein assay kit (Thermo Scientific, Waltham, MA, USA, Cat. No. 23235), following all manufacturer’s instructions.

### 2.2. HDL Treatments

A single pooled sample of isolated HDL was divided into 64 aliquots and treated with various reagents to simulate processes observed in chronic disease. HDL aliquots were first diluted into 0.5 mg/mL protein concentration with 1× PBS (Thermo Fisher, Waltham, MA, USA, Cat. No. 10010023). Detailed treatment reagents and conditions are reported in Table 1. Briefly, to simulate the effects of oxidative stress, HDLs were incubated with copper (II) sulfate (CuSO_4_, 10 μM, 18 h), hydrogen peroxide (H_2_O_2_, 160 µM, 2 h), or hypochlorous acid (HOCl, 160 µM, 2 h); to simulate an acidic pH environment, HDLs were incubated in pH 5.5 ammonium acetate buffer (20 mM, 18 h); to induce glycation and fructosylation, processes which occur in conditions of hyperglycemia and/or high fructose intake, HDLs were incubated in 50 mM glucose for 120 h, or in 100 mM fructose solution for 192 h; to simulate alterations in glycosylation, HDLs were incubated with the N-glycan removal enzyme peptide:N-glycosidase F (PNGase F, 25,000 U/mL, 4 h), or the sialic acid removal enzyme α2-3, 6, 8, 9 neuraminidase A (sialidase, 2000 U/mL, 2 h). To explore the effects of antioxidant carotenoid molecules on HDL particle structure and function, HDL samples were incubated with lutein (1 mM, 18 h) or zeaxanthin (1 mM, 18 h). All treatments were incubated at 37 °C in a water bath. Because different treatments had different incubation time requirements, time-based control samples were generated for each incubation time (2, 4, 18, 120, or 192 h) without any treatment. An additional HDL aliquot that received 0 h of any treatment (i.e., frozen right after isolation) served as a master control. Each set of treatments and controls was performed in 4 replicates. In total, 64 samples were generated (10 treatment groups, 5 time-control groups, and 1 master control group, with 4 replicates for each group). The excessive reagent was removed by 5 repeats of filtration through a 50 kDa filter (Amicon Ultra-4 50 kDa, MilliporeSigma, Burlington, MA, USA, Cat. No. UFC805024) followed by dilution with 1× PBS (Thermo Fisher, Waltham, MA, USA, Cat. No. 10010023).

To investigate the preventative potential of lutein/zeaxanthin on induced oxidation of HDL, 30 µM lutein or zeaxanthin was first incubated with HDL at 4 °C for 2 h. The excessive reagent was then removed from the treated HDL solution, as described above, followed by treatment with 160 µM H_2_O_2_ at 37 °C for 2 h to introduce oxidation. After oxidation treatment, the excessive reagent was removed, as described above. Control samples with HDL only, with HDL + H_2_O_2_, and with lutein or zeaxanthin only were prepared for background adjustments. All samples were prepared in triplicate.

### 2.3. Glycosylation Modification Efficacy Determinations

The glycosylation modification of HDL samples was confirmed by analyzing the glycan composition from treated vs. control HDL (Supplemental Figures S1 and S2) using methods described previously [21]. Briefly, the HDLs were resuspended in 100 µL of 5 mM dithiothreitol in 100 mM ammonium bicarbonate solution and denatured in a boiling water bath for 2 min. After the samples were cooled to room temperature, 2 µL of PNGase F was added. The samples were heated to 37 °C using a microwave (CEM Corporation, Matthews, NC, USA) at 20 watts for 10 min to release the glycans and incubated in a 37 °C water bath overnight to hydrolyze the primary amines of the released glycans to hydroxyl groups. After the incubation, 350 µL of nanopure water was added. The samples were ultracentrifuged at 200,000× *g* for 45 min at 4 °C, and the supernatant containing the N-glycans was desalted using a porous graphitic carbon (PGC) solid phase extraction plate. The plate was conditioned using 80% (*v*/*v*) acetonitrile in water with 0.1% (*v*/*v*) trifluoroacetic acid and equilibrated using nanopure water. The samples were loaded and washed using nanopure water before eluted using 40% (*v*/*v*) acetonitrile in water with 0.05% (*v*/*v*) trifluoroacetic acid. The eluates were vacuum-dried prior to mass spectrometry analysis.

The samples were reconstituted in nanopure water and analyzed using an Agilent 6520 Accurate-Mass Q-TOF LC/MS equipped with a PGC microfluidic chip (Agilent Technologies, Santa Clara, CA, USA). The glycans were separated with a binary LC gradient using solvents (A) 3% (*v*/*v*) acetonitrile in water with 0.1% (*v*/*v*) formic acid, and (B) 90% (*v*/*v*) acetonitrile in water with 1% (*v*/*v*) formic acid at a flow rate of 300 nL/min: 0–2 min, 0–0% B; 2–20 min, 0–16% B; 20–40 min, 16–72% B; 40–42 min, 72–100% B; 42–52 min, 100–100% B; 52–54 min, 100–0% B; 54–65 min, 0–0% B. MS spectra were scanned from *m*/*z* 600 to 2000 in positive ionization mode. The most abundant precursor ions in each MS1 spectrum were selected for fragmentation through collision-induced dissociation (CID) based on the equation V_collision_ = 1.8 × (*m*/*z*) ÷ (100 V − 2.4 V). Glycans were extracted by searching the data against an in-house human N-glycan database using the Agilent Mass Hunter Qualitative Analysis software (B.08.00) with a mass tolerance of 20 ppm. The relative abundances of glycans were compared using their chromatographic peak areas.

### 2.4. Lutein/Zeaxanthin Incorporation Confirmation

The confirmation of lutein/zeaxanthin incorporation into HDL particles was conducted using methods described previously [24,34,35] with modifications. Lutein or zeaxanthin was incubated with 0, 50, or 500 (for lutein only) µg/mL HDL at 37 °C for 2 h, and excessive lutein/zeaxanthin in the solution was removed, as described in Section 2.2. The absorption of samples at 445 nm was measured on a Synergy H1 plate reader (BioTek, Winooski, VT, USA). All samples were prepared in triplicate. Lutein/zeaxanthin incorporation was confirmed (Supplemental Figure S3).

### 2.5. Negative-Stain Transmission Electron Microscopy and Image Analysis

Particle size average and distribution were assessed using negative-stain transmission electron microscopy (NS-TEM, Talos L120C, FEI, Thermo Fisher, MA, USA) and the image analysis software ImageJ (version 1.52v) [36], as described previously [32]. Briefly, 5 µL of HDL sample at 50 µg/mL protein concentration in 1XPBS was loaded on a glow-discharged carbon-coated grid (TedPella Inc., Redding, CA, USA, Cat. No. 01840-F). The sample was let sit on the grid for 1 min for sample binding and then removed using filter paper. Five µL of 2% uranyl formate stain was then loaded on the grid and quickly removed using filter paper. The staining steps were repeated 4 more times. After the last stain was removed, the grid was allowed to air-dry in a dark environment before it was stored until imaging. During sample imaging, samples were viewed using a Talos L120C electron microscope at 80 kV HT and 36,000× magnification. Sample images were captured with a defocus of 1.5–2.0 nm and an exposure time of 300 ms with a 4096 × 4096 resolution. At least 10 images were captured for each sample. The particle size of samples was analyzed using the image analysis tool ImageJ with a customized IJM script. Briefly, images were adjusted for threshold using the “Default” mode. Particles with an area less than 19.625 nm^2^ (diameter less than 5 nm) were selected using the “Particle Analysis” function and removed. Particles between 38.465 nm^2^ and 706.5 nm^2^ (diameter between 7 nm and 30 nm) were selected. These selected particles were then filtered with geometric parameters, excluding particles that had a circularity < 0.5, roundness < 0.5, and aspect ratio > 1.5. Particles that were not excluded were used for particle size data analysis. The median particle diameter was calculated for each experiment replicate. Particle diameter distribution was described as the percentage of particles at each 1-nm size group between 7 nm and 15 nm diameter divided by total particle number within the 7 nm–15 nm diameter range.

### 2.6. CEC Assay

The CEC of control and treatment samples were measured in mouse macrophages J774A.1 (ATCC, Manassas, VA, USA, Cat. No. TIB-67) using a commercially available CEC assay kit (Abcam, Cambridge, UK, Cat. No. ab19685), following the manufacturer’s instructions with minor adjustment, as described previously [37]. Briefly, about 50,000 J774 macrophages were loaded onto each well in a 96-well microplate (Sigma, MO, USA, Cat. No. CLS3603) and incubated at 37 °C, 5% CO_2_, and 90% relative humidity for 18 h in RPMI 1640 medium (Thermo Fisher, MA, USA, Cat. No. 11875093) with 1% penicillin/streptomycin (Thermo Fisher, MA, USA, Cat. No. 15140122), and 10% fetal bovine serum (Thermo Fisher, MA, USA, Cat. No. A3160502). After incubation, the medium was discarded, and cells were washed with serum-free RPMI 1640 medium 3 times. Cells were then incubated with BODIPY-labeled cholesterol, acyl-coenzyme A: cholesterol acyltransferase inhibitor, and cyclic adenosine monophosphate provided by the assay kit at 37 °C, 5% CO_2_, and 90% relative humidity for 4 h for cholesterol loading. After cholesterol loading, the remaining medium was removed, and cells were washed with serum-free RPMI 1640 medium 3 times, followed by the addition of 10 µg (total protein) cholesterol acceptors (HDL samples or plasma as the quality control) and positive control and negative control provided by the assay kit. The cells were then incubated at 37 °C, 5% CO_2_, and 90% relative humidity for 4 h for cholesterol efflux. After cholesterol efflux incubation, the supernatant portion was collected and transferred to a new microplate. The remaining cell pellet was lysed with M-PER cell lysis buffer (Thermo Fisher Scientific, Waltham, MA, USA, Cat. No. 78505) for 30 min. The fluorescence of the supernatant and the lysed fraction were measured at 482/515 nm (excitation/emission) on a Synergy H1 plate reader (BioTek, Winooski, VT, USA). The CEC was calculated by the fluorescence value of the supernatant fraction divided by the sum of the fluorescence value of the supernatant and the lysed fractions. To account for the inter-plate variability, CEC values were normalized to the value of the plasma sample control on each plate to adjust for inter-plate variability, as previously described, resulting in a CEC index [38].

### 2.7. LCAT Activity Assay

A commercially available kit (Roar Biomedical, New York, NY, USA, Cat. No. mak107) was used to measure the LCAT activity of 5 µg HDL (total protein) following the manufacturer’s instructions, using a Synergy H1 plate reader (Agilent Technologies, Santa Clara, CA, USA) at excitation = 340 nm, and at emission at 390 nm or 470 nm. The ratio between fluorescence emission at 390 nm to 470 nm was calculated as a measurement of LCAT activity. The higher the ratio, the higher the LCAT activity. LCAT activity values were normalized to the value of a plasma sample control on each plate to adjust for inter-plate variability, as previously described, resulting in an LCAT activity index [38].

### 2.8. PON1 Activity Assay

The Ca^2+^-dependent PON1 activity of HDL was determined by monitoring the conversion of paraoxon to 4-nitrophenol over time at 405 nm [13,39]. HDL samples (10 µg, total protein) were placed into a 96-well plate and mixed thoroughly with 200 µL buffer at pH 8 containing Tris (100 mM) (Sigma, MO, USA, Cat. No. T1503), CaCl_2_ (2 mM) (Sigma, MO, USA, Cat. No. C4901), and paraoxon (1 mM) (Sigma, MO, USA, Cat. No. 36186). The absorbance of light at 405 nm wavelength was measured every 5 min on a plate reader. Kinetic plots were made for each group by plotting absorbance at 405 nm against measurement timepoints. The linear regions of the kinetic plots were determined, and the difference between the beginning and the end absorbance values within the linear regions were found. The difference was then divided by the time span between when the two absorbance points were measured and further divided by the molar extinction coefficient of the product 4-nitrophenol (17,100 L·mol^−1^·cm^−1^). PON1 activity of samples was expressed as nM 4-nitrophenol/minute. PON1 activity values were normalized to the value of a plasma sample control on each plate to adjust for inter-plate variability, as previously described, resulting in a PON1 activity index [38].

### 2.9. Statistical Analysis

Mean particle diameter, percentage of particle number at each 1-nm size group, CEC index, LCAT 390/470 ratio, and PON1 activity for each replicate were tested for outliers using the Grubb’s Test for Outliers with an α = 0.05. Mean particle diameter, percentage of particle number at each size group, CEC index, LCAT 390/470 ratio, and PON1 activity for each treatment were compared to the control group that corresponded to their treatment times using a two-sample *t*-test with significance value reported as *p* < 0.05, assuming equal variance. For multigroup comparisons, differences between groups were tested with one-way ANOVA, and pair-wise comparisons were conducted using post hoc Tukey’s HSD. Statistically significant findings are indicated as * *p* < 0.05, ** *p* < 0.01, *** *p* < 0.001, and **** *p* < 0.0001. The normality of the outcome variables was inspected using the Shapiro–Wilks test. The equal variance between groups was examined using Levene’s test.

## 3. Results

### 3.1. HDL Particle Size and Distribution

**Figure 1:**
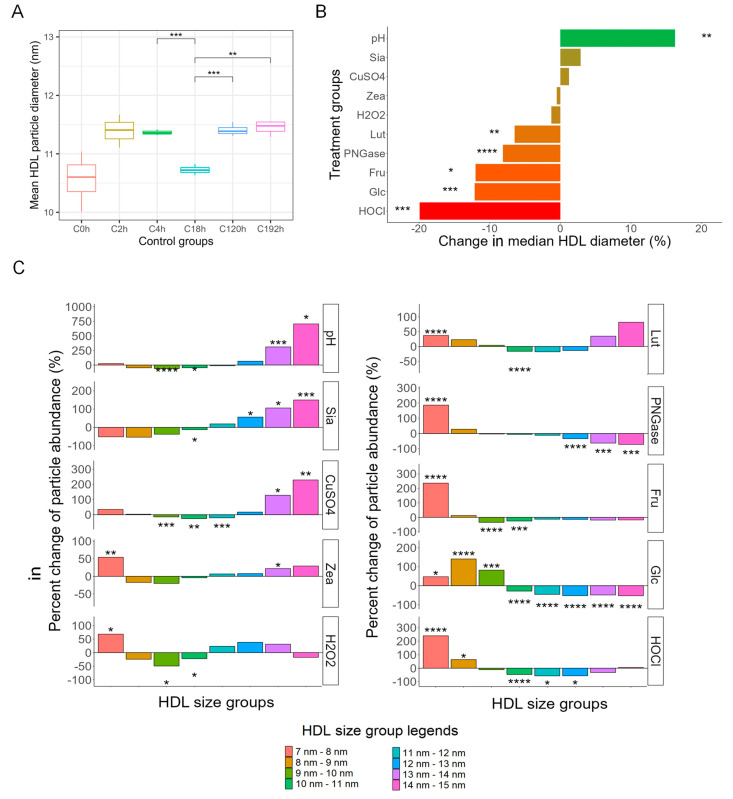
Diameter changes in HDL in different treatment groups compared to corresponding time controls. (**A**) Comparisons of HDL diameter in different time control groups. (**B**) Change in median HDL particle diameter measured on transmission electron microscope images of HDL samples. (**C**) Analysis of HDL 1-nm size group abundance change in response to treatments. Subfraction abundance was calculated as 100% × (abundance of HDL subfraction in the treatment group) ÷ (abundance of HDL subfraction in the corresponding time-control group). Statistical significance is shown as * (*p* < 0.05), ** (*p* < 0.01), *** (*p* < 0.005), and **** (*p* < 0.001). Treatments legend: HOCl = hypochlorous acid; Glc = glucose; Fru = fructose; PNGase = PNGase F; Lut = lutein; H_2_O_2_ = hydrogen peroxide; Zea = zeaxanthin; CuSO_4_ = copper (II) sulfate; Sia = α-2, 3, 6, 8-neuraminidase; pH = pH 5.5.

Median HDL particle size and particle size distribution were assessed to determine structural changes in response to the treatments. The HDL diameter results in the time controls were generally similar (Figure 1A), except for C18h (10.7 nm ± 0.0818 nm), which was significantly smaller than that of C4h (11.4 nm ± 0.0483 nm), C120h (11.4 nm ± 0.101 nm), and C192h (11.5 nm ± 0.124 nm). Figure 1B shows that HDL samples that were incubated with HOCl (9.12 nm ± 0.11 nm vs. 11.39 nm ± 0.28 nm, −19.93%, *p* < 0.005), glucose (10.02 nm ± 0.35 nm vs. 11.41 nm ± 0.10 nm, −12.13%, *p* < 0.005), fructose (10.07 nm ± 0.35 nm vs. 10.73 nm ± 0.08 nm, −12.01%, *p* < 0.05), PNGase F (10.44 nm ± 0.06 nm vs. 11.37 nm ± 0.05 nm, −8.13%, *p* < 0.001), and lutein (10.03 nm ± 0.17 nm vs. 10.73 nm ± 0.08 nm, −6.48%, *p* < 0.01) had significantly smaller median particle diameter than their respective control groups. HDL treated with an acidic pH of 5.5 was the only treatment group that had a significantly larger (12.46 nm ± 0.55 nm vs. 10.73 ± 0.08 nm, +16.23%, *p* < 0.01) median diameter than control. HDL treated with H_2_O_2_, zeaxanthin, CuSO_4_, and sialidase did not have significantly different particle sizes from their control groups (Figure 1B).

Because HDL particles are structurally heterogeneous and have a continuous size range, the size distribution of HDL in different treatment groups was further characterized and compared to their control groups (Figure 1C). The population of particles within the diameter range of 7 nm–15 nm was divided by 1-nm increment into eight subclasses (7 nm–8 nm, 8 nm–9 nm, etc.). For each treatment group, the abundance of particles within each subclass was compared to the same subclass in the corresponding control group. Fructose, glucose, H_2_O_2_, HOCl, lutein, PNGase F, and zeaxanthin treatment resulted in a significant increase in small particles compared to their corresponding control groups (Figure 1C). For the glucose and PNGase F groups particularly, a shift in particle size distribution from larger to smaller particles was observed. In contrast, CuSO_4_, acidic pH, and sialidase shifted particle size distribution from smaller to larger particles (Figure 1C).

### 3.2. HDL CEC, LCAT Activity and PON1 Activity after Direct Treatments

**Figure 2:**
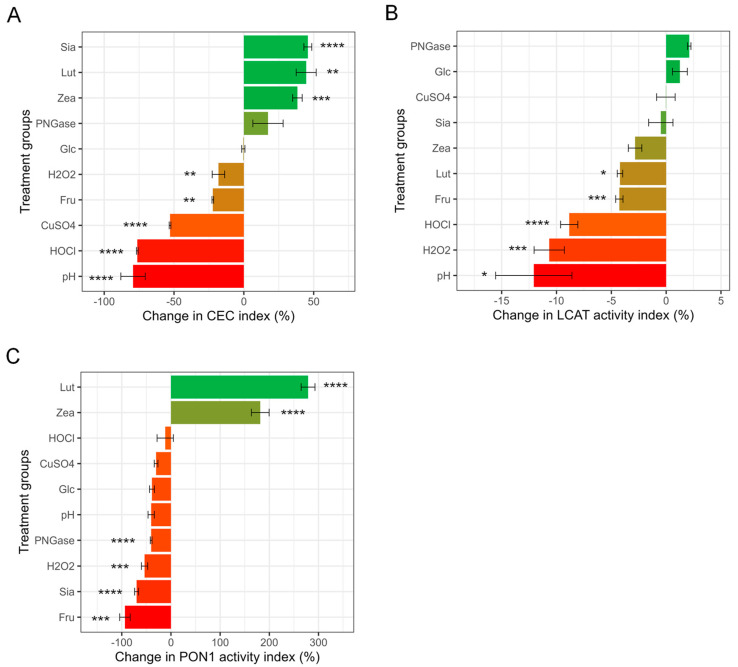
Percentage change in HDL functional measurements. (**A**) Change in CEC. CEC was measured using a commercially available kit in J774A.1 mouse macrophage cells. Change in CEC was calculated as 100% × (CEC of treatment groups − CEC of corresponding time-control group) ÷ (CEC of corresponding time-control group). (**B**) Percent LCAT activity change. LCAT activity was measured as the sample fluorescent emission ratio at λ = 390 nm over λ = 470 nm using commercially available kit at excitation λ = 340 nm. Change in LCAT activity was calculated as 100% × (LCAT activity of treatment groups − LCAT activity of corresponding time-control group) ÷ (LCAT activity of corresponding time-control group). (**C**) Percent PON1 activity change. PON1 enzyme activity was reported as the nM of paraoxon converted to 4-nitrophenol per minute. Change in PON1 activity was calculated as 100% × (PON1 activity of treatment groups − PON1 activity of corresponding time-control group) ÷ (PON1 activity of corresponding time-control group). Statistical significance is shown as * (*p* < 0.05), ** (*p* < 0.01), *** (*p* < 0.005), **** (*p* < 0.001). Treatments legend: HOCl = hypochlorous acid; Glc = glucose; Fru = fructose; PNGase = PNGase F; Lut = lutein; H_2_O_2_ = hydrogen peroxide; Zea = zeaxanthin; CuSO_4_ = copper (II) sulfate; Sia = α-2, 3, 6, 8-neuraminidase; pH = pH 5.5.

The control-normalized mean HDL CEC index among treatment groups was significantly different (ANOVA *p* < 0.001) after correction to their corresponding time controls. Pairwise comparison showed that the pH treatment group had the lowest HDL CEC value (0.193 ± 0.163, significantly lower than Glc, Lut, PNGase, Sia, and Zea groups), followed by the HOCl group (0.217 ± 0.0115, significantly lower than CuSO_4_, Fru, Lut, Sia, and Zea groups), the CuSO_4_ group (0.443 ± 0.0115, significantly lower than Fru, Lut, Sia, and Zea groups), the H_2_O_2_ group (0.750 ± 0.0700, significantly lower than Lut, Sia, and Zea groups), and the Fru group (0.797 ± 0.0114, significantly lower than Glc and Sia groups). The Glc group (0.953 ± 0.0981) was significantly higher than the pH group. The PNGase (1.25 ± 0.232), Zea (1.29 ± 0.0586), Sia (1.34 ± 0.0537), and Lut (1.35 ± 0.135) groups had the highest HDL CEC values (Table S1). We further compared the percentage of change in CEC in the treatment groups compared to their time controls (Figure 2A). Low pH (0.11 ± 0.095 vs. 0.58 ± 0.04, −81%, *p* < 0.001), HOCl (0.13 ± 0.01 vs. 0.61 ± 0.04, −78%, *p* < 0.001), CuSO_4_ (0.26 ± 0.01 vs. 0.58 ± 0.04, −56%, *p* < 0.001), H_2_O_2_ (0.46 ± 0.044 vs. 0.61 ± 0.043, −25%, *p* < 0.01), and fructose (0.44 ± 0.0058 vs. 0.58 ± 0.041, −21%, *p* < 0.01) treatment resulted in significantly lower CEC (Figure 2A). In contrast, zeaxanthin (0.75 ± 0.032 vs. 0.58 ± 0.043, +29%, *p* < 0.005), sialidase (0.82 ± 0.03 vs. 0.61 ± 0.04, +34%, *p* < 0.001), and lutein (0.79 ± 0.078 vs. 0.58 ± 0.043, +35%, *p* < 0.01) treatment resulted in significantly higher CEC than their control groups (Figure 2A).

Control-normalized LCAT activity was not significantly different among treatment groups (ANOVA *p* = 0.08), and none of the treatment groups was significantly different from one another after adjustment for multiple pairwise comparisons (Table S1). Nevertheless, several treatments significantly changed HDL LCAT activity compared to their time controls: low pH (0.88 ± 0.070 vs. 1.0 ± 0.023, −12%, *p* < 0.05), H_2_O_2_ (0.91 ± 0.024 vs. 1.0 ± 0.024, −11%, *p* < 0.005), HOCl (0.93 ± 0.016 vs. 1.0 ± 0.024, −8.8%, *p* < 0.001), fructose (0.93 ± 0.0056 vs. 0.97 ± 0.015, −4.3%, *p* < 0.05), and lutein treatment (0.96 ± 0.0035 vs. 1.0 ± 0.023, −4.2%, *p* < 0.05) reduced LCAT activity compared to their controls, whereas none of the other treatments altered LCAT activity (Figure 2B).

Control-adjusted PON1 activity was significantly different among treatment groups (ANOVA < 0.001). The Fru group had the lowest normalized PON1 activity value (0.060 ± 0.14) and was significantly lower than the Lut and Zea groups, followed by the Sia group (0.31 ± 0.072, significantly lower than the CuSO_4_, Lut, and Zea groups), the H_2_O_2_ group (0.46 ± 0.12, significantly lower than Lut and Zea groups), the PNGase group (0.58 ± 0.038, significantly lower than Lut and Zea groups), the Glc group (0.61 ± 0.071, significantly lower than Lut and Zea groups), the pH group (0.65 ± 0.12, significantly lower than Lut and Zea groups), the CuSO_4_ group (0.76 ± 0.082, significantly lower than Sia, Lut and Zea groups), and the HOCl group (0.88 ± 0.33, significantly lower than Lut and Zea groups). The Zea (3.1 ± 0.40) and Lut (4.1 ± 0.31) groups had the highest normalized PON1 activity values among the treatment groups (Table S1). Paraoxon hydrolysis activity (i.e., PON1 activity) of HDL treated with fructose (1.2 nM/minute vs. 1.9 nM/minute, −94%, *p* < 0.005), sialidase (1.6 nM/minute vs. 5.1 nM/minute, −69%, *p* < 0.001), H_2_O_2_ (2.4 nM/minute vs. 5.1 nM/minute, −54%, *p* < 0.005), and PNGase F (3.0 nM/minute vs. 5.3 nM/minute, −42%, *p* < 0.001) was significantly reduced compared to the corresponding time-control groups (Figure 2C). Samples treated with zeaxanthin (9.9 nM/minute vs. 3.5 nM/minute, +182%, *p* < 0.001) and lutein (13 nM/minute vs. 3.5 nM/minute, +279%, *p* < 0.001), on the other hand, showed a significant and substantial increase in PON1 activity (Figure 2C). Variation in functional assay measurements of the control groups is shown in Supplemental Figure S4.

### 3.3. HDL CEC, LCAT Activity, and PON1 Activity after Lutein/Zeaxanthin Pre-Incubation Followed by Oxidation Treatment

**Figure 3:**
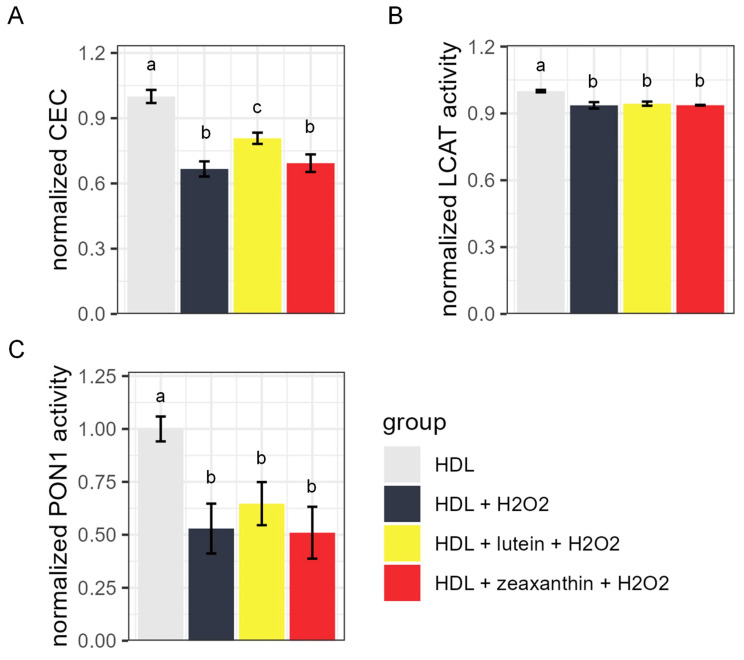
Functional measurement of HDL with and without carotenoid pre-treatment. (**A**) Normalized cholesterol efflux capacity (CEC), (**B**) normalized lecithin–cholesterol acyltransferase (LCAT) activity, and (**C**) normalized paraoxonase-1 (PON1) activity of HDL particles without H_2_O_2_ oxidant treatment (light grey), with H_2_O_2_ treatment but without carotenoid pre-incubation (dark grey), with lutein pre-treatment followed by H_2_O_2_ treatment (yellow), and with zeaxanthin pre-treatment followed by H_2_O_2_ treatment (red). For all groups with the same letter above the bars, the difference between the means was not statistically significant.

To further explore whether lutein and zeaxanthin can prevent HDL from oxidation, we performed additional experiments to oxidize HDL samples after lutein/zeaxanthin pre-incubation. Figure 3 shows the CEC, LCAT activity, and PON1 activity of HDL samples after H_2_O_2_-induced oxidation with or without lutein/zeaxanthin pre-incubation. H_2_O_2_ treatment reduced the CEC, LCAT, and PON1 activity of HDL (Figure 3A–C). Pre-incubating HDL with 30 µM lutein for 2 h (0.81 ± 0.026, Figure 3A, yellow bar) attenuated the effect of H_2_O_2_ (0.67 ± 0.034, Figure 3A, gray bar) on CEC reduction, but the attenuation of PON1 activity loss did not reach statistical significance (Figure 3C). Neither lutein nor zeaxanthin pre-incubation had an effect on LCAT activity loss (Figure 3B). Pre-incubating HDL with 30 µM zeaxanthin for 2 h did not have a significant preventative effect on HDL CEC or PON1 activity loss (Figure 3A,C red vs. dark gray column).

## 4. Discussion

The aim of this study was to determine the effects of multiple chemical modifications often observed in pathological processes on HDL particle size distribution and functional properties, and to determine the effects of the carotenoids lutein and zeaxanthin on the same parameters. The antioxidant carotenoids lutein and zeaxanthin consistently improved HDL CEC and PON1 activity, and lutein treatment slightly reduced LCAT activity. Lutein and zeaxanthin are transported primarily by HDL in the body [26] and exert well-characterized functions in the eye [40]. High lutein supplementation in animals has been found to reduce oxidized low-density lipoprotein (LDL) [41]. Moderate lutein supplementation in the form of egg yolk or milk in human trials has been shown to increase plasma lutein levels without increasing HDL-C or LDL-cholesterol [42]. On the other hand, oxidants, including HOCl, H_2_O_2_, and CuSO_4_, reduced HDL function. The CEC results are in agreement with previous findings, where oxidizing agents were shown to impair ATP-binding cassette transporter A1-mediated cholesterol transport [17] as well as other CEC-related components [43]. Treating human subjects with γ-radiolysis water containing hydroxyl radical and peroxide anions significantly reduced PON1 activity and the sulfhydryl groups on PON1 [44]. Besides lutein, LCAT activity was reduced by low pH, H_2_O_2_, HOCl, and high-concentration fructose incubation. The mechanism of such reductions is not clear. One possibility could be the removal of LCAT from its complex with HDL through these treatments. These results suggest that the CEC, LCAT, and PON1 activity of HDL are sensitive to oxidative stress.

The most surprising result was that high glucose was the only treatment that did not affect any of the HDL functions, whereas high fructose treatment consistently reduced all three functional assays of HDL. It is possible that functional differences could be observed with longer incubation times [45]. However, longer incubation times introduce HDL degradation, which may confound the effects of glycation. High blood glucose and fructose levels are commonly found in patients with T2DM or in people who habitually consume sugary food or beverages [46,47]. Most of the previous studies on the adverse effects of high fructose diet revolve around its interaction with body cells and the lipogenic effect of fructose [48,49]. These results indicate that high fructose may be more destructive to HDL function than a high glucose environment.

Low pH treatment also had a strong negative effect on HDL function, especially CEC and LCAT activity. Previous studies with similar experimental designs found that low pH increased the CEC of HDL [23]. However, instead of neutralizing pH by adding a base, we filtered out the low pH solution using a filtering unit with 50 kDa pore size and reconstituted HDL with PBS. This procedure can remove free apoA-I, a 28 kDa protein that contributes significantly to CEC, from the sample. This may be what caused the differences between our results and previous findings. The LCAT and PON1 activity of low pH-treated HDL was also reduced compared to their respective control, though the difference in PON1 activity did not quite reach statistical significance (*p* = 0.07). A simple explanation may be that at low pH, the proteins unfolded, and some of the free apoA-I, LCAT, and PON1 from the HDL detached from the particle [23]. Both LCAT (~47 kDa) and PON1 (~43 kDa) are smaller than the pore size of our filtering unit and, thus, likely did not remain in the HDL sample as free proteins.

The effects of glycosylation modification, sialidase, and PNGase F treatments on HDL functions were more unpredictable. While sialidase treatment increased CEC, it reduced PON1 activity. PNGase F treatment also decreased PON1 activity, but it appeared to increase CEC. Terminal sialic acid residues have an array of important roles, including modification of the microenvironment, masking of the underlying glycans, and facilitating specific recognition and interactions, for example, via lectin receptors [50]. It is currently unknown what aspects of HDL-associated sialylation could have led to the observed increase in CEC. On the other hand, both sialidase and PNGase F treatment reduced HDL PON1 activity. PON1 is a glycoprotein [51], so it is possible that glycosylation removal reduces its activity. Another possibility is that sialic acids themselves may be naturally modified into uncharged lactones between the C-1 carboxyl group and hydroxyl groups from adjacent sugars [50], which then may be an additional substrate for PON1, and thus the control group would contain more sialic acid lactone than the sialic acid-free sialidase treatment group, resulting in lower apparent PON1 activity in the sialidase-treated group. Interestingly, although LCAT is also a glycosylated protein [52], LCAT activity was not significantly affected by either sialidase or PNGase F treatment.

The structural changes in HDL after each treatment were characterized by their change in particle diameter and subclass abundance. HOCl treatment resulted in a smaller median HDL particle diameter in this study. Several key structural components of HDL, including apoA-I [53], apoA-II [53], and apoE [54], undergo structural changes after oxidation. It is less clear why H_2_O_2_ treatments, also a potent oxidizing agent, did not result in reduced median HDL particle diameter. One possible explanation is that HOCl is a more powerful oxidant than H_2_O_2_ [17]. In this study, since the amount of HOCl and H_2_O_2_ were in equal molar ratios, the oxidizing power of H_2_O_2_ may not be strong enough to induce a significant change in median HDL size that is comparable to the effects of HOCl. In fact, the changes in HDL subgroups in the H_2_O_2_ treated samples were partially comparable to that of the HOCl groups; both treatments increased the abundance of the smallest 7 nm–8 nm particles and reduced the 10 nm–11 nm medium-size HDL particles, though both magnitude and statistical significance were lower for the H_2_O_2_ treatment group. Low pH substantially increased median HDL particle size, with the largest HDL (13 nm–15 nm) specifically being increased in abundance compared to the control (Figure 1). On the other hand, Cu^2+^ treatment changed HDL subfraction distribution in the same way as low pH, but the overall median HDL size was not different compared to the control group. Such differences were expected. Cu^2+^-treated HDL has been shown to be slightly larger by HPLC analysis but not obvious enough to be seen under TEM [55], and also had reduced CEC [14,55], which agrees with our observations. For an acidic pH environment, previous studies with a similar design have shown that compared to the elution profile of nontreated HDL3 particles, which eluted as one peak, HDL incubated at pH 5.5 buffer showed two separate peaks, one larger and the other smaller. The smaller peak was identified as a free apoA-I protein [23].

We also observed that the majority of treatments that reduced LCAT activity (lutein, high fructose, HOCl, H_2_O_2_, and acidic pH) also reduced the median HDL particle diameter. This is in agreement with historical reports that LCAT is enriched in large HDL in proteomic studies [32]. This observation suggests that either there is a structural preference of the LCAT protein for large HDL particles or that removing LCAT from HDL through external factors results in reduced HDL maturation.

Lutein incubation reduced median HDL diameter and, more specifically, increased the abundance of the smallest HDL subfraction while reducing the medium-size HDL subfraction. Zeaxanthin treatment achieved similar subfraction changes, but the overall median HDL diameter was unchanged. The incorporation of lipophilic molecules may disturb the structure of HDL and potentially introduce cleavage of a large HDL into more than one HDL particle or fragment [56]. We further showed that pre-incubating HDL with 30 µM lutein (physiological level) attenuated the negative effects of H_2_O_2_-induced oxidation on CEC by 21% (Figure 3A). On the other hand, zeaxanthin pre-incubation did not protect HDL particle function from H_2_O_2_-induced oxidation. It is important to note that lutein and zeaxanthin, among other carotenoids, are extensively modified, undergoing isomerization and other modifications during the process of digestion and absorption, and also have multiple interactions with gut microbes [57], which could all have important implications for the effects of these molecules in vivo.

Glucose and fructose treatments both reduced median HDL diameter. There is limited information on HDL particle size after glucose or fructose treatments. Patients with type 2 diabetes have been shown to have decreased average HDL diameter, specifically, a shift from medium and large HDL particles to small HDL particles [58,59,60]. However, this shift in HDL particle size distribution in type 2 diabetes may be related to metabolic effects associated with the well-known mechanism of cholesterol ester transfer protein (CETP)-mediated HDL metabolism by hepatic lipase [61].

PNGase F and sialidase treatments modify the glycan structure of HDL-associated proteins. PNGase F treatment reduced median HDL diameter. N-glycans alter the conformational preferences of proteins to a higher probability of more compact and stable conformations [62,63]. However, the impacts of glycosylation on protein conformation and association with HDL particles, as well as the downstream effects on HDL particle structure, have not been characterized. It is possible that the lipid–protein complex was loosened in response to N-glycan removal, and a larger HDL may thus disintegrate into more than one smaller HDL. Our results support this hypothesis since the largest HDLs (12 nm–15 nm) were significantly decreased while the smallest HDLs (7 nm–8 nm) were significantly increased in abundance (Figure 2) after PNGase F treatment. In contrast, sialidase treatment did not significantly alter median HDL size. However, sialidase treatment significantly increased the abundance of the largest HDL subfractions (12 nm–15 nm), while the smaller subfractions were slightly but not significantly reduced. Sialidase specifically removes terminal sialic acid residues from glycan chains. Without sialic acids, proteins of HDL particles that are known to be sialylated (e.g., apoA-II, apoCs, apoD, apoE, apoJ, and apoM) [64] may adopt different conformations, affecting the structure and function of HDL particles.

## 5. Conclusions

In this study, we found that incubation with lutein and zeaxanthin improved several HDL functions, whereas various oxidants, low pH, and high fructose concentration were the most deleterious treatments that consistently induced HDL functional loss. Specifically, oxidants and fructose reduced both the CEC and LCAT activity of HDL particles, which are involved in the efflux of cholesterol from cells and HDL particle maturation, respectively. These findings suggest that exposure to oxidizing agents, low pH, and high fructose concentrations would be particularly deleterious to the process of reverse cholesterol transport. On the other hand, the removal of sialic acid and incubation with lutein and zeaxanthin increased the CEC of HDL particles. The carotenoid antioxidants lutein and zeaxanthin also greatly improved HDL PON1 activity by as much as 200–300%. Our findings demonstrate that exposure to factors that are often found in environments where inflammatory processes are taking place (i.e., pro-oxidant molecules, low pH) and high fructose concentrations are harmful to HDL particle function and alter particle size distribution, whereas incubation with antioxidant carotenoids lutein and zeaxanthin improves HDL functionality. These findings have important implications for the management of diseases in which loss of HDL function is related to disease pathophysiology.

## Figures and Tables

**Table 1:** Detailed characteristics of experimental treatment groups. All treatments were performed at 37 °C with pH 7.4 phosphate-buffered saline (except for the acidic pH treatment).

Treatment Name/Purpose	Treatment Reagent (Vendor)	Concentration	Treatment Time (h)	Reference
CuSO_4_/oxidation	copper (II) sulfate (CuSO_4_) (Cat. #: C988L31, Neta Scientific, Marlton, NJ, USA)	10 µM	18	[14]
H_2_O_2_/oxidation	hydrogen peroxide (H_2_O_2_) (Cat. #: H1065, Spectrum Chemical, Gardena, CA, USA)	160 µM	2	[17]
HOCl/oxidation	hypochlorous acid (HOCl) (Cat. #: S1316, Spectrum Chemical, Gardena, CA, USA)	160 µM	2	[17]
Acidic pH/acidification	ammonium acetate buffer, pH 5.5 (Cat. #: 40100184-1, Spectrum Chemical, Gardena, CA, USA)	20 mM	18	[23]
Glucose/glycation	Glucose (Cat. #: 40700008-1, Spectrum Chemical, Gardena, CA, USA)	50 mM	120	[20]
Fructose/fructosylation	Fructose (Cat. #: 40600008-1, Spectrum Chemical, Gardena, CA, USA)	100 mM	192	[33]
PNGase F/deglycosylation	PNGase F (Cat. #: P0705S, New England Biolabs, Ipswich, MA, USA)	25,000 U/mL	4	[21]
Sialidase/desialylation	α2-3, 6, 8, 9 neuraminidase A (Cat. #: P722S, New England Biolabs, Ipswich, MA, USA)	2000 U/mL	2	[21]
Lutein/antioxidation	Lutein (Cat. #: PHR1699, Sigma, St. Louis, MO, USA).	1 mM	18	[24]
Zeaxanthin/antioxidation	Zeaxanthin (Cat. #: 1733119, Sigma, St. Louis, MO, USA)	1 mM	18	[24]

## Data Availability

Data are contained within the article and Supplementary Materials.

## Supplementary Materials

The following supporting information can be downloaded at: https://www.mdpi.com/article/10.3390/antiox13050616/s1.
