# Supplementary material for: Lutein and Zeaxanthin Enhance, Whereas Oxidation, Fructosylation, and Low pH Damage High-Density Lipoprotein Biological Functionality
Source: Antioxidants (Basel). 2024 May 18;13(5):616. doi: 10.3390/antiox13050616 (PMC11118252; doi:10.3390/antiox13050616)
Supplement: Supplementary file 1 [file antioxidants-13-00616-s001.zip › antioxidants-2819382-supplementary.pdf]

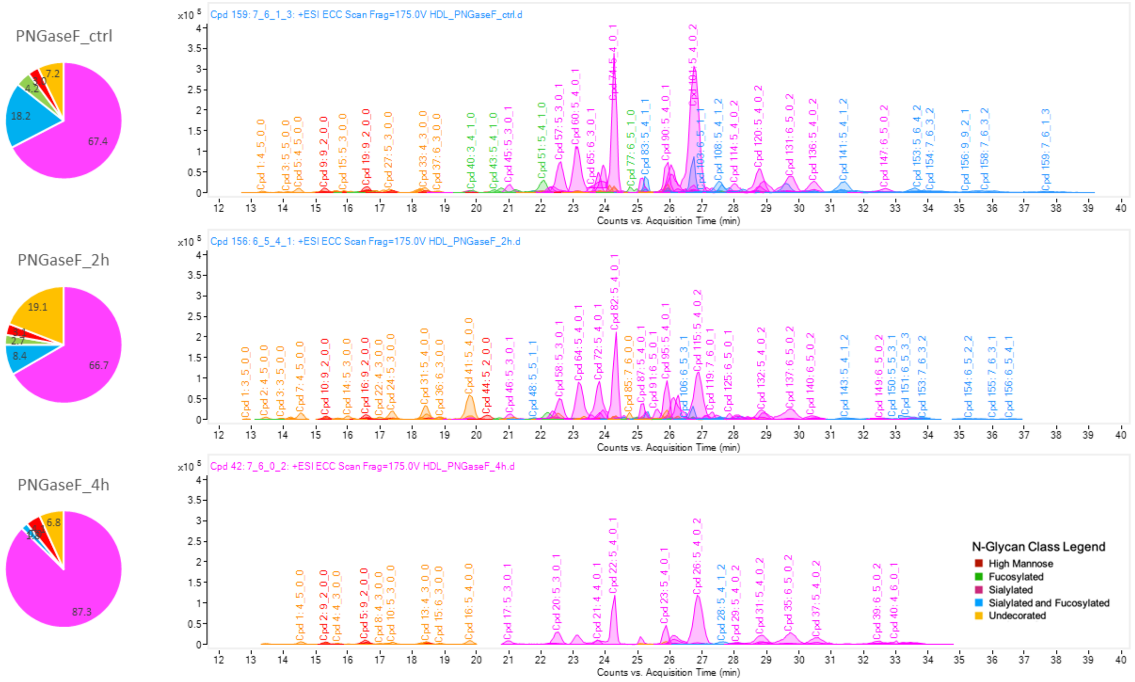

Figure S1. Glycoprofile of HDL samples incubated with PNGase F for different amounts of time. HDL isolates were treated with PNGase F (25,000 U/mL) for 0, 2, or 4 hours. The extracted compound chromatograms show the released N-glycans annotated with glycan compositions (extracted compound number followed by the numbers of hexose_N-acetylhexosamine_fucose_sialic acid). The peaks are color coded to show N-glycan subtypes according to the legend: red, high mannose; green, fucosylated; pink, sialylated; blue, fucosylated and sialylated; orange, undecorated. The pie charts represent the relative abundances of each N-glycan subtype. The chromatograms show a depletion of all types of glycans after PNGase F treatment for 2h and especially 4h.

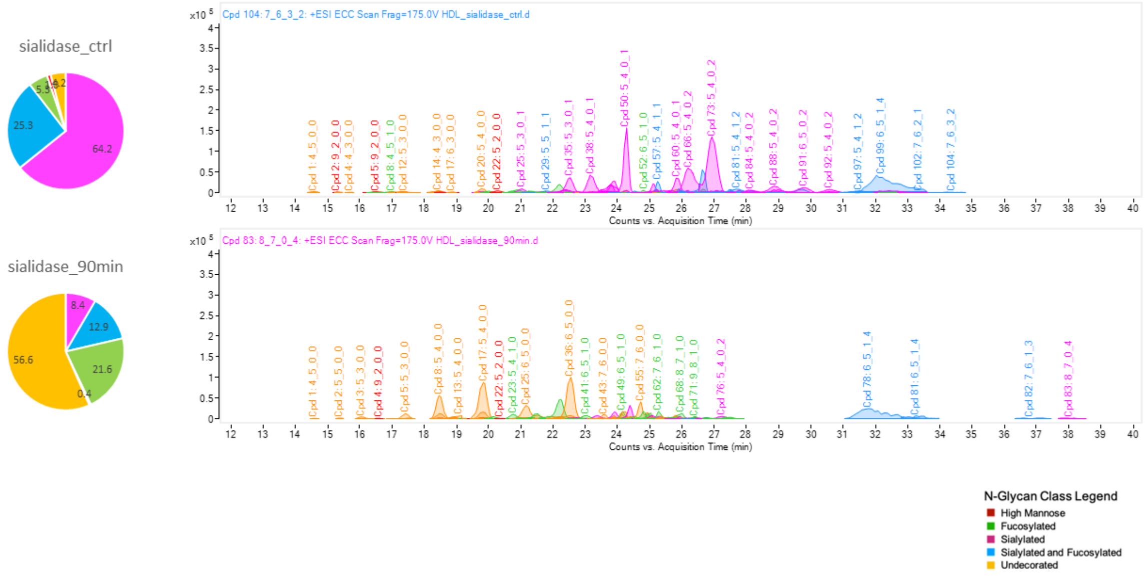

Figure S2. Glycoprofile of HDL samples incubated with sialidase (α2-3, 6, 8, 9 neuraminidase A) for different amounts of time. HDL isolates were treated with sialidase (2,000 U/mL) for 0 or 90 minutes. The extracted compound chromatograms show the released N-glycans annotated with glycan compositions (extracted compound number followed by the numbers of hexose_N-acetylhexosamine_fucose_sialic acid). The peaks are color coded to show N-glycan subtypes according to the legend: red, high mannose; green, fucosylated; pink, sialylated; blue, fucosylated and sialylated; orange, undecorated. The pie charts represent the relative abundances of each N-glycan subtype. The chromatograms show a depletion of sialylated (pink) as well as sialylated and focyslated (blue) glycans after neuraminidase treatment for 90 min.

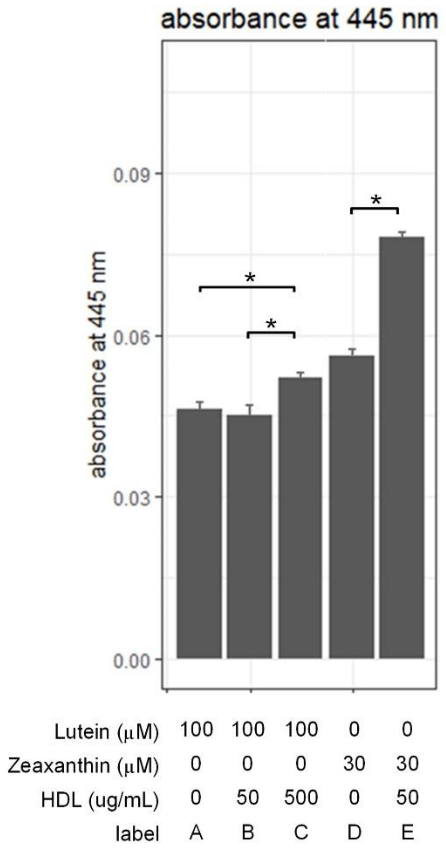

Figure S3. Comparisons of solution absorbance at 445 nm. Solution with 100 μM lutein (A, B, C) incubated with 0 (A), 50 (B), and 500 (C) μg/mL HDL and solution with 30 μM zeaxanthin (D, E) incubated with 0 (D) or 50 (E) μg/mL HDL were measured for absorbance at 445 nm wavelength after solvent replacement to evaluate retainment of carotenoid molecules as a reflection of carotenoid incorporation in HDLs.

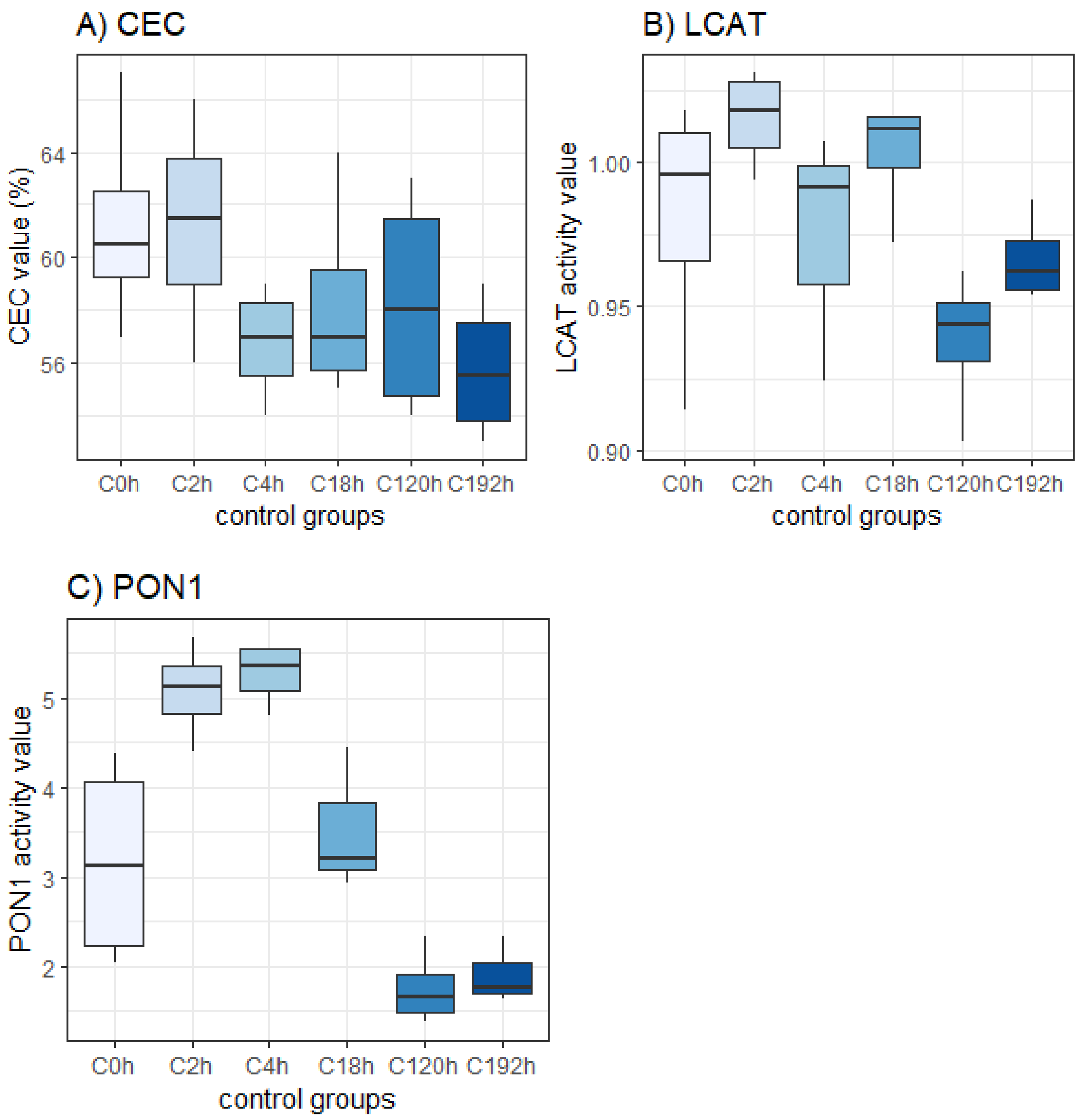

Figure S4. Comparisons of HDL functional assay values of Control groups. The A) cholesterol efflux capacity (CEC), B) lecithin-cholesterol acyltransferase (LCAT) activity, and C) paraoxonase-1 (PON1) activity of the control group (C0h) and various time-control groups (C2h, C4h, C18h, C120h, and C192h).

Table S1. Differences in time control-normalized CEC, LCAT, and PON1 activity among treatment groups. Shown are the means and standard deviations (SD) for cholesterol efflux capacity (CEC), lecithin cholesterol acyltransferase (LCAT) activity, and paraoxonase 1 (PON1) activity for each treatment group normalized to corresponding time-control. ANOVA analysis was performed to determine whether there were statistically significant differences among the treatment groups. The p-values for the ANOVA analyses were: $p < 0.001$ for CEC, $p = 0.002$ for LCAT, and $p < 0.001$ for PON1. Pairwise comparisons between treatment groups were performed using a t-test with the Benjamini–Hochberg adjustment for CEC, LCAT, and PON1. Treatment groups that were not statistically significantly different were labeled with the same letter in the comparisons column of each biological assay. Treatments legends: **CuSO4** = copper (II) sulfate; **Fru** = fructose; **Glc** = glucose; **H2O2** = hydrogen peroxide; **HOCl** = hypochlorous acid; **Lut** = lutein; **pH** = pH 5.5; **PNGase** = PNGase F; **Sia** = α-2, 3, 6, 8-neuraminidase; **Zea** = zeaxanthin.

**Variables**	**CEC**			**LCAT**			**PON1**		
	**mean**	**s.d.**	**comparisons**	**mean**	**s.d.**	**comparisons**	**mean**	**s.d.**	**comparisons**
**CuSO4**	0.443	0.012	a	0.985	0.0071	ns	0.758	0.0818	a
**Fru**	0.797	0.012	b	0.943	0.0289	ns	0.0600	0.141	ab
**Glc**	0.953	0.098	abc	1.02	0.0115	ns	0.610	0.0707	abc
**H2O2**	0.750	0.0700	abcd	0.923	0.0551	ns	0.463	0.115	abcd
**HOCl**	0.217	0.0115	cde	0.910	0.0200	ns	0.883	0.328	abce
**Lut**	1.35	0.135	bcf	0.973	0.0231	ns	4.14	0.309	f
**pH**	0.193	0.163	abde	0.880	0.0698	ns	0.650	0.121	abcdeg
**PNGase**	1.25	0.232	abcdefg	0.997	0.0231	ns	0.575	0.0379	abcdefgh
**Sia**	1.34	0.0537	cfgh	0.993	0.0153	ns	0.313	0.0723	bcdefgh
**Zea**	1.29	0.0585	bcfgh	0.987	0.0306	ns	3.08	0.395	f
